# Pathways for outpatient management of venous thromboembolism in a UK centre

**DOI:** 10.1186/s12959-016-0120-2

**Published:** 2016-12-05

**Authors:** Robin Condliffe

**Affiliations:** Pulmonary Vascular Disease Unit, Sheffield Teaching Hospitals NHS Foundation Trust, Sheffield, UK

**Keywords:** Deep vein thrombosis, Oral anticoagulant, Patient pathway, Pulmonary embolism, Venous thromboembolism

## Abstract

It has become widely recognised that outpatient treatment may be suitable for many patients with venous thromboembolism. In addition, non-vitamin K antagonist oral anticoagulants that have been approved over the last few years have the potential to be an integral component of the outpatient care pathway, owing to their oral route of administration, lack of requirement for routine anticoagulation monitoring and simple dosing regimens.

A robust pathway for outpatient care is also vital; one such pathway has been developed at Sheffield Teaching Hospitals in the UK. This paper describes the pathway and the arguments in its favour as an example of best practice and value offered to patients with venous thromboembolism.

The pathway has two branches (one for deep vein thrombosis and one for pulmonary embolism), each with the same five-step process for outpatient treatment. Both begin from the point that the patient presents (in the Emergency Department, Thrombosis Clinic or general practitioner’s office), followed by diagnosis, risk stratification, treatment choice and, finally, follow-up.

The advantages of these pathways are that they offer clear, evidence-based guidance for the identification, diagnosis and treatment of patients who can safely be treated in the outpatient setting, and provide a detailed, stepwise process that can be easily adapted to suit the needs of other institutions. The approach is likely to result in both healthcare and economic benefits, including increased patient satisfaction and shorter hospital stays.

## Background

Historically, patients diagnosed with deep vein thrombosis (DVT) and pulmonary embolism (PE) have been treated as inpatients owing to the potential for serious complications, including death. In recent years it has been recognised that many patients with acute DVT may be safely treated in the outpatient setting. Furthermore, it is possible to identify patients with acute PE who are at low risk of deterioration and may also be suitable for ambulatory management or early discharge [1–4].

Appropriate outpatient management of DVT and PE may be beneficial to patients and the healthcare system alike. Potential benefits include improvements in patient satisfaction and reduced healthcare costs associated with a shorter hospital stay. Limited data are available to compare these outcomes and further research is needed [5]. Non-vitamin K antagonist (VKA) oral anticoagulant (NOAC) therapy may provide benefits for patient management in ambulatory care compared with low molecular weight heparin (LMWH) overlapping with, and followed by, a VKA [6]. NOAC therapy involves oral administration, no routine coagulation monitoring requirements, a single-drug approach (with rivaroxaban and apixaban) and fewer follow-up appointments [6].

One potential disadvantage of ambulatory care is that opportunities for follow-up, patient education and communication between primary and secondary care may be lost if a patient is discharged from hospital without an adequate protocol in place. Healthcare professionals (HCPs) at Sheffield Teaching Hospitals have developed a patient pathway for venous thromboembolism (VTE) management to improve the transition of patients from hospital to home. This pathway has proved effective in ensuring adequate follow-up and communication between all HCPs involved. The development of such a pathway can also help streamline processes and clinical decision-making, improving efficiency and ensuring consistent high-quality care. This article presents the Sheffield VTE management pathways for DVT and PE as examples of best practice, demonstrating their value in VTE management, and discusses the benefits of NOAC use in ambulatory care.

### Venous thromboembolism management

In the UK, the management of DVT varies widely. A recent UK audit reported a lack of coordinated services in this area and called for standardised and consistent protocols [7]. The Sheffield pathway is an evidence-based pathway, developed by the whole VTE management team, in which low-risk patients may be treated in an ambulatory care setting, while patients at higher risk are admitted to hospital. This approach also reduces the associated burden on healthcare resources and patients’ time. A treatment pathway also provides clarity in an area with a large choice of diagnostic tools, an increasing number of treatment options and various forms of presentation (e.g. provoked or unprovoked, mild, moderate or severe symptoms). In the past, many hospitals had an uncoordinated VTE management strategy with a range of diagnostic assessment, treatment, and patient follow-up pathways, depending on which department the patient presented to [8, 9]. Optimal VTE management includes rapid assessment, diagnosis and treatment; patient information and support; and follow-up. Follow-up allows clinical improvement to be confirmed, chronic complications to be monitored and an optimal anticoagulation approach to be planned.

### The Sheffield venous thromboembolism management pathway: deep vein thrombosis

Typically, a patient may enter the Sheffield DVT pathway in one of three ways. A patient may: 1) present directly to the Emergency Department and be transferred to the Thrombosis Clinic (open during working hours); 2) visit their general practitioner and be referred to the Thrombosis Clinic/Emergency Department; or 3) present as an inpatient (e.g. in the instance of a post-operative venous thromboembolic event). These three entry levels involve contact with several hospital HCPs, including nurses, VTE specialist nurses, junior doctors, pharmacists and consultants (collectively, the multidisciplinary team [MDT]). The type of VTE diagnosed and the patient’s medical history determine which members of the MDT are involved in each individual patient pathway, including longer-term follow-up.

### Step 1: Patient presentation

**Fig. 1:**
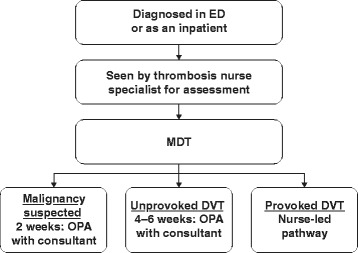
Sheffield deep vein thrombosis pathway. DVT, deep vein thrombosis; ED, Emergency Department; MDT, multidisciplinary team; OPA, outpatient appointment

When a patient presents with suspected DVT, a general medical history and physical examination will be conducted; if DVT is considered likely, the patient will enter the DVT pathway (Fig. 1). If a specialist nurse in the Thrombosis Clinic is not available, an Emergency Department physician will assess the patient.

### Step 2: Diagnosis in the thrombosis clinic (or by emergency department physician)

The validated two-level DVT Wells’ score indicates whether a DVT is likely or unlikely based on the patients clinical signs, symptoms and through exclusion of other causes [10]. A score of ≥2 indicates that DVT is likely; a score of ≤1 indicates that DVT is unlikely (Table 1) [10]. The likelihood of DVT can be further determined by a blood test for D-dimer, a degradation product of a blood clot. D-dimer levels are typically elevated in patients with an acute VTE [11]. However, a negative D-dimer result is more clinically important in order to ‘rule out’ DVT, because a positive result can arise in conditions other than DVT [11].

**Table 1:** Deep vein thrombosis Wells’ score [10]

Criteria	Points
Active cancer	+1
Paralysis, paresis or recent plaster cast of the lower limb	+1
Bedridden for 3+ days or major surgery within 12 weeks	+1
Pain/tenderness along deep vein system	+1
Swollen leg	+1
Calf swelling >3 cm more than asymptomatic leg	+1
Pitting oedema in symptomatic leg only	+1
Collateral superficial veins	+1
History of DVT	+1
Alternative cause is considered at least as likely as DVT	−2
Outcome:	
DVT unlikely:	Score ≤1 (consider trauma, cellulitis)
DVT likely:	Score ≥2

*DVT* deep vein thrombosis

If DVT is considered a likely diagnosis, the patient will be sent for an ultrasound scan, preferably on the same day. If the ultrasound scan is scheduled for the following day or after a weekend, immediate anticoagulation with a LMWH injection is administered.

### Step 3: Risk stratification

At the point of diagnosis, and when considering DVT treatment options, each patient must be assessed for complications and frailty. This may determine the treatment type, level of observation required and whether treatment can be safely administered at home. Most DVT cases can be managed safely at home, but for certain patients, for example if the event is post-operative or if the patient is at high risk of falling, a hospital stay may be required. Certain patients with proximal iliofemoral DVT may be candidates for catheter-directed thrombolysis. Risk of bleeding events within the first 3–6 months of anticoagulation may be assessed using the HAS-BLED (Hypertension, Abnormal renal and liver function, Stroke, Bleeding, Labile international normalised ratios, Elderly, Drugs or alcohol) [12] or the RIETE (based on recent major bleeding, creatinine >1.2 mg/mL, anaemia, cancer, clinically overt PE and age >75 years) risk scores [13]. The HAS-BLED score was derived from patients receiving anticoagulation for atrial fibrillation. The utility of these studies in assessing early risk of bleeding is limited.

### Step 4: Treatment strategy

In Sheffield, until recently, the majority of patients diagnosed with DVT were treated initially with LMWH while warfarin therapy was commenced. Patients receiving warfarin require routine coagulation monitoring to ensure that they stay within the therapeutic range, evaluated with the international normalised ratio [14]. Several other choices of anticoagulation are now available in Europe, with the NOACs apixaban, dabigatran, edoxaban and rivaroxaban approved for the treatment of acute DVT and PE [15–18]. These therapies do not require routine coagulation monitoring and have all been shown to be non-inferior to warfarin in terms of VTE recurrence [19–22].

To improve clinician familiarity and hence patient safety, we have elected to use a single NOAC for the initial treatment of VTE. In the Sheffield DVT pathway the majority of DVT cases are managed using rivaroxaban if patients have a creatinine clearance ≥30 mL/min, unless contraindicated. This oral, single-drug approach – 15 mg twice daily for the first 21 days and then 20 mg once daily for longer-term treatment – is a simple regimen that facilitates the majority of patients being treated at home [15]. Other NOACs are considered after initial anticoagulation on a case-by-case basis.

### Step 5: Follow-up

An outpatient appointment with the thrombosis nurse at the Thrombosis Clinic is arranged for all patients undergoing outpatient management, approximately 21 days after the initial DVT event. This aligns with when the rivaroxaban dose, if rivaroxaban is the prescribed drug, is changed to 20 mg once daily. The patient is provided with education about their anticoagulation therapy, including the importance of adherence to treatment, warning signs for bleeding, symptoms of recurrent VTE and when to contact a HCP. The patient is also provided with a contact number for the Thrombosis Clinic if they need to access more information.

For patients with an unprovoked DVT, in which the cause of DVT is unclear, an outpatient appointment with a consultant haematologist is arranged to discuss long-term therapy. In patients for whom malignancy is suspected, an outpatient appointment is booked within 2 weeks of initial presentation to discuss options for further investigations or scans. Thrombophilia testing may also be arranged in selected patients. The initial treatment duration with rivaroxaban is 3 months, and longer-term treatment is discussed when appropriate.

### The Sheffield venous thromboembolism management pathway: pulmonary embolism

#### Step 1: Patient presentation

**Fig. 2:**
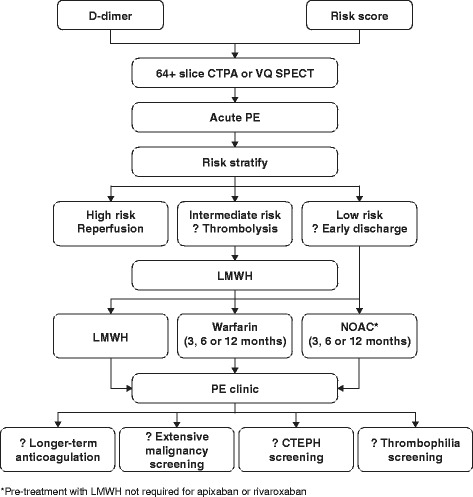
Sheffield pulmonary embolism pathway. CTEPH, chronic thromboembolic pulmonary hypertension; CTPA, computed tomography pulmonary angiogram; LMWH, low molecular weight heparin; OAC, oral anticoagulant; PE, pulmonary embolism; VQ SPECT, ventilation/perfusion single-photon emission tomography [35]

Patients may present with symptoms indicative of an acute PE either to their general practitioner (leading to referral to the Emergency Department) or directly to the Emergency Department, where they enter into the PE pathway (Fig. 2).

#### Step 2: Diagnosis in the thrombosis clinic (or by emergency department physician)

The two-level Wells’ PE score is used to determine whether PE is a likely or unlikely diagnosis [23]. The Wells’ PE score – both the full and simplified versions – has been validated for use in clinical settings [24, 25]. The score includes clinical signs and symptoms of DVT, PE as the most likely diagnosis, heart rate >100 bpm, recent immobilisation or surgery, previous VTE, haemoptysis and active or previous malignancy (Table 2) [23]. If the simplified Wells’ score suggests that PE is likely, the patient proceeds to diagnostic imaging, most commonly computed tomography pulmonary angiogram, with ventilation/perfusion single-photon emission computed tomography being reserved for patients with significant renal dysfunction, contrast allergy or pregnancy. If the simplified Wells’ score suggests that PE is unlikely, D-dimer levels are used to identify patients in whom diagnostic imaging is not required. Although withholding of anticoagulation in patients with levels below an age-adjusted D-dimer threshold (age in years × 10) was demonstrated to be associated with a very low risk of subsequent VTE, these data have not been validated in other populations [26]. Therefore, the current approach is to use a standard threshold of <500 ng/mL to exclude acute PE in patients with a simplified Wells’ score of ≤4 [1].

**Table 2:** Simplified pulmonary embolism Wells’ score [23, 25]

Clinical feature	Original score	Simplified score
Clinical signs and symptoms of DVT (minimum of leg swelling and pain with palpation of the deep veins)	3	1
An alternative diagnosis is less likely than PE	3	1
Heart rate ≥100 beats per minute	1.5	1
Immobilisation (for >3 days) or surgery in the previous 4 weeks	1.5	1
Previous DVT/PE	1.5	1
Haemoptysis	1	1
Active cancer	1	1
Outcome		
PE unlikely:	Score ≤4	Score 0 or 1
PE likely:	Score >4	Score ≥2

*DVT* deep vein thrombosis, *PE* pulmonary embolism

#### Step 3: Risk stratification

Following diagnosis of acute PE, patients undergo risk assessment for early deterioration. Patients with low blood pressure (<90/60 mmHg) and/or signs of clinical shock (high-risk patients) should be considered for immediate reperfusion therapy, most commonly with systemic thrombolysis [1]. Non-high-risk patients may be further categorised into intermediate- and low-risk groups based on a combination of risk score and markers of right ventricular dysfunction and ischaemia [1].

**Fig. 3:**
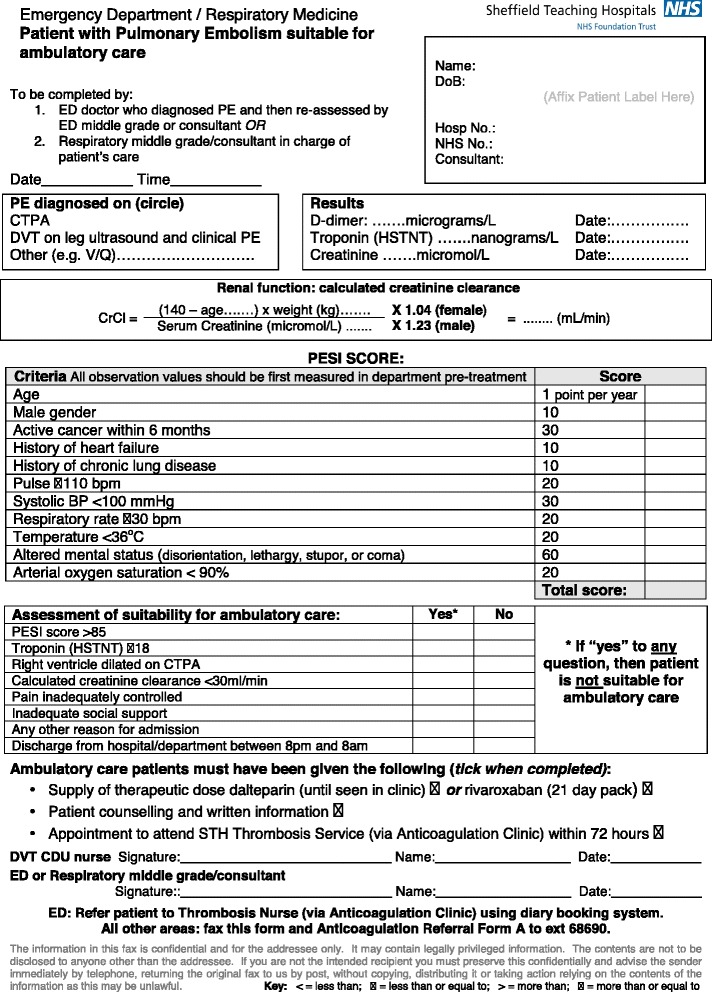
Patient assessment form

The PESI and the sPESI are the two most validated clinical–physiological risk scoring systems (Table 3). Patients with a PESI class I–II or sPESI score of 0 are considered low risk (<3% risk of deterioration) and may be considered for outpatient management [1]. Aujesky et al. performed the largest randomised controlled trial of outpatient PE management to date and demonstrated that patients with PESI class I or II, who also did not meet certain exclusion criteria (Table 3), were not put at increased risk by early discharge [2]. If markers of right ventricular dysfunction or ischaemia (e.g. N-terminal of the prohormone brain natriuretic peptide or high-sensitivity troponin) are also negative, the risk of early PE-related deterioration is <1% [27, 28]. It is unclear whether these additional biomarkers should be a mandatory addition to the PESI or sPESI for identifying patients who can be considered for discharge. Although these additional tests may improve safety, this may be at the expense of the number of patients who would qualify for outpatient management. The HESTIA criteria provide an alternative approach to risk stratification, incorporating several clinical, practical and social issues (Table 4) [3]. The HESTIA study showed that the absence of any of these criteria could safely identify patients for outpatient management of PE [3]. On closer inspection, the HESTIA criteria are actually very similar to the exclusion criteria employed in the study by Aujesky et al. [2] (Table 4). Because the PESI and sPESI currently have more data supporting their use in risk stratification across the whole spectrum of patients with acute PE, the current protocol therefore incorporates PESI scoring in all patients diagnosed with acute PE (Fig. 3). The majority of social and practical exclusion criteria used by Aujesky et al. [2] have been incorporated. Currently, patients in our centre are also required to have a normal-sized RV on CTPA to fulfil criteria for outpatient management. It is possible that the criteria may become less conservative in the future in light of recent data and changing guidelines. For example, the HESTIA investigators observed that the presence of RV dilatation did not increase risk related to outpatient management, assuming that no HESTIA criteria were met [3].

**Table 3:** PESI and sPESI scores [33, 34]

Prediction factors	PESI	sPESI
Age >80 years	Age in years	1
Male gender	+10	-
Cancer	+30	1
Heart failure	+10	1^a^
Chronic lung disease	+10	
Pulse ≥110 beats/minute	+20	1
Systolic blood pressure <100 mmHg	+30	1
Respiratory rate ≥30 breaths/minute	+20	-
Temperature <36 °C	+20	-
Altered mental status	+60	-
Arterial oxyhaemoglobin saturation <90%	+20	1
Outcome		
Low risk:	Class I: ≤65 Class II: 66–85	PESI = 0
Intermediate risk:	Class III: 86–105	
High risk:	Class IV: 106–125 Class V: >125	PESI = ≥1

*PESI* Pulmonary Embolism Severity Index, *sPESI* simplified Pulmonary Embolism Severity Index

^a^Single combined category of chronic cardiopulmonary disease

**Table 4:** Comparison of HESTIA criteria and exclusion criteria used by Aujesky et al. [2, 3]

HESTIA criteria: Zondag [3]	Exclusion criteria: Aujesky [2]
Is the patient haemodynamically unstable?	SBP <100 mmHg
Is thrombolysis or embolectomy necessary?	
>24 h oxygen to maintain sats >90%	Oxygen saturation <90%
Active bleeding or high risk of bleeding	Active bleeding High risk of bleeding (stroke within the preceding 10 days, GI bleed within the last 14 days or platelet count <75,000/mm^3^)
PE diagnosed on anticoagulation?	Therapeutic anticoagulation (INR ≥2.0) at diagnosis
Severe pain needing IV pain medication for >24 h	Chest pain needing opiates
Medical or social reason for treatment in hospital (infection, malignancy, no support system)	Barriers to treatment adherence or follow-up
CrCl <30 mL/min	Severe renal failure (CrCl <30 mL/min)
Severe liver impairment	
Documented history of HIT	HIT
Is the patient pregnant?	
	Obesity (weight >150 kg)

*CrCl* creatinine clearance, *GI* gastrointestinal, *HIT* heparin-induced thrombocytopenia, *INR* international normalised ratio, *IV* intravenous, *PE* pulmonary embolism, *SBP* systolic blood pressure

Patients without hypotension but with PESI class III–V or sPESI class >0 are at intermediate risk of early deterioration and require hospital admission. Patients in this group who have both radiological evidence of right ventricular dysfunction (from CTPA or echocardiography, if performed) and elevated plasma biomarkers (BNP, NT-proBNP or troponin) are at intermediate-high risk of deterioration; this group require especially close monitoring and consideration for reperfusion therapy if there is evidence of further deterioration [1]. Other features, such as the presence of DVT on compression ultrasonography [29] or elevated lactate levels [30], may be useful in further refining identification of intermediate-to-high risk patients at particular risk of deterioration.

#### Step 4: Treatment strategy

Patients at high risk of early deterioration should undergo reperfusion therapy, most commonly with systemic thrombolysis, although catheter-directed therapy and surgical embolectomy will sometimes be necessary if there are significant contraindications to systemic thrombolysis. Patients at low or intermediate risk of deterioration are candidates for either LMWH/VKA, or a NOAC. We would generally treat patients at intermediate-high risk – in whom subsequent thrombolysis may potentially be necessary – with LMWH and a VKA. Patients at low risk of deterioration are considered for outpatient management. Although currently published studies regarding outpatient management of acute PE have utilised LMWH and VKA, the practical benefits of NOACs (especially the NOACs rivaroxaban and apixaban, which do not require pre-treatment with LMWH) make them an attractive method of anticoagulation in patients undergoing outpatient management. This role of rivaroxaban in outpatient PE management is currently being investigated in more detail in the multicentre HoT-PE study [31]. In intermediate-risk patients who are admitted, reassessment of PESI or sPESI score after 48 h may identify patients now suitable for early discharge and outpatient management [1].

#### Step 5: Follow-up

If patients undergo outpatient management, they are reviewed within 48 h by the VTE nurse specialist. The patient’s clinical state is assessed to ensure no clinical deterioration. Results of initial malignancy screening are reviewed, including a focused history and examination, review of blood results and urinalysis. Dependent on the results of these tests, further tests may be arranged. The current anticoagulation method is reviewed and a plan for ongoing anticoagulation is made in conjunction with the patient. If the patient is treated with rivaroxaban, a second appointment is made for approximately 21 days after diagnosis which coincides with the change in dosing from 15 mg twice daily to 20 mg once daily.

Education and counselling are important components of patient care. At the time of PE diagnosis, an individual treatment plan will be provided and treatment options will be discussed. Later, outpatient appointments help to ensure patients understand the reasons behind why a PE occurred, the recommended treatment and why treatment adherence is important. It is also an opportunity for the patient to be fully reassured and for any questions or concerns to be discussed.

In Sheffield, we review patients at approximately 3 months following their acute PE at a consultant-led, combined respiratory-haematology clinic. The patient’s initial history and radiological investigations are reviewed to confirm the diagnosis, the nature of the event (i.e. provoked or unprovoked) and to assess the likelihood of chronic complications. A proportion of patients with ongoing, new breathlessness will undergo further investigation (often a combination of echocardiography, nuclear perfusion scanning and/or computed tomography pulmonary angiogram) to assess for the presence of chronic thromboembolic pulmonary hypertension. Plans regarding ongoing anticoagulation management are then made. Longer-term anticoagulation is considered following unprovoked events, whereas anticoagulation can often be stopped after 3 months following strongly provoked clots. In selected patients thrombophilia testing may be indicated, while D-dimer level testing after withdrawing anticoagulation may further refine estimates of the risk of recurrence in selected patients with partially provoked events [32].

## Conclusion

The Sheffield VTE management pathways for DVT and PE are examples of best practice within the UK. These pathways facilitate the smooth transition of patients from hospital to home, while maintaining regular patient follow-up. VTE management for many patients with distal DVT, proximal DVT or low-risk PE can be safely carried out as part of ambulatory care, particularly with the involvement of specialist anticoagulation nurses and the use of NOACs. Use of a pathway similar to the Sheffield VTE pathway may reduce the burden on secondary care and the length of hospital stays. Patient satisfaction may also increase with same-day diagnosis, shorter hospital stay, fewer injections, and follow-up in the same thrombosis service.

## Abbreviations

CTEPH: Chronic thromboembolic pulmonary hypertension
CTPA: Computed tomography pulmonary angiogram
DVT: Deep vein thrombosis
HAS-BLED: Hypertension Abnormal renal and liver function, Stroke, Bleeding, Labile international normalised ratio, Elderly, Drugs or alcohol
HCP: Healthcare professional
HoT-PE: Home treatment of pulmonary embolism
LMWH: Low molecular weight heparin
MDT: Multidisciplinary team
NOAC: Non-vitamin K antagonist oral anticoagulant
OAC: Oral anticoagulant
OPA: Outpatient appointment
PE: Pulmonary embolism
PESI: Pulmonary Embolism Severity Index
sPESI: Simplified Pulmonary Embolism Severity Index
VKA: Vitamin K antagonist
VQ SPECT: Ventilation/perfusion single-photon emission tomography
VTE: Venous thromboembolism
